# Efecto clínico y metabólico de una intervención multidisciplinaria en el marco de un programa de atención integral para niños y adolescentes con obesidad

**DOI:** 10.7705/biomedica.4593

**Published:** 2020-03-30

**Authors:** Nora Alejandra Zuluaga, Adriana Osorno, Alba Lozano, Óscar Villada

**Affiliations:** 1 Programa de Obesidad Infantil, Hospital Universitario de San Vicente Fundación, Medellín, Colombia Hospital Universitario de San Vicente Fundación Medellín Colombia; 2 Dirección de Investigaciones, Hospital Universitario de San Vicente Fundación, Medellín, Colombia Hospital Universitario de San Vicente Fundación Medellín Colombia

**Keywords:** síndrome metabólico, índice de masa corporal, obesidad, factores de riesgo, niño, adolescentes, ejercicio, dieta saludable, educación, Metabolic syndrome, body mass index, obesity, risk factors, children, adolescents, exercise, healthy diet, education

## Abstract

**Introducción.:**

La obesidad infantil requiere una atención con programas multidisciplinarios que integren todas sus dimensiones.

**Objetivo.:**

Describir los cambios clínicos y metabólicos en pacientes con obesidad después de su participación en un programa de atención integral en obesidad infantil.

**Materiales y métodos.:**

Se hizo un estudio observacional y analítico retrospectivo de una cohorte de pacientes de 6 a 17 años de edad atendidos en el programa de obesidad del Hospital Universitario de San Vicente Fundación (2012-2015), el cual incluyó la atención multidisciplinaria y una intervención educativa. Se evaluaron variables antropométricas y de laboratorio en el momento del ingreso al programa y en la última evaluación. Se exploraron las diferencias según el tiempo de seguimiento.

**Resultados.:**

Se evaluaron 53 pacientes con una edad promedio de 11 ± 2 años, 52,8 % de los cuales eran hombres. El seguimiento de los pacientes fue de 18 ± 6 meses, aunque en el 30 % de ellos fue de 31 a 36 meses. Se encontró una disminución del índice de masa corporal (IMC; puntaje Z) entre el ingreso (2,75 ± 0,58) y el último control (2,32 ± 0,63) con un valor de p de 0,000 (IC_95_% 0,27-0,58). El 79,25 % de los pacientes redujo el puntaje Z del IMC. Esta disminución fue significativa independientemente del tiempo de seguimiento. La proporción de pacientes con un puntaje Z del IMC mayor de 3 pasó del 33,4 al 14,6 %. El número de criterios positivos para el síndrome metabólico disminuyó en el seguimiento. Los niveles de triglicéridos y de hemoglobina A1c (HbA1c) mejoraron significativamente.

**Conclusiones.:**

El manejo de la obesidad infantil con una intervención multidisciplinaria asociada a apoyo educativo grupal continuo puede influir significativamente en los cambios clínicos y metabólicos. Es necesario prolongar el tiempo de seguimiento para prevenir las recaídas.

## Abstract (en)

**Introduction:**

The approach to childhood obesity requires multidisciplinary programs including all the dimensions susceptible to management.

**Objective:**

To describe the clinical and metabolic changes in patients with obesity after a comprehensive care program for childhood obesity.

**Materials and methods:**

We conducted a retrospective observational and analytical study in a cohort of patients between 6 and 17 years old treated in the obesity program at the *Hospital Universitario San Vicente Fundación* (2012-2015). We carried out multidisciplinary care and educational intervention. Anthropometric and laboratory variables were evaluated both at admission to the program and in the last evaluation and statistical differences were sought according to the follow-up time.

**Results:**

We evaluated 53 patients, of whom 52.8% were men, with an average age of 11 ± 2 years. The follow-up extended for 18 ± 6 months though 30% of the patients were followed for 31 to 36 months. There was a decrease in the BMI (Z score) between admission (2.75 ± 0.58 and the last control (2.32 ± 0.63) with a p-value of 0.000 (95% CI: 0.27 -0.58); 79.25% of the patients reduced the BMI Z score. This decrease was significant regardless of the follow-up time. The proportion of patients with a BMI Z score >3 decreased from 33.4% to 14.6%. The number of positive criteria for metabolic syndrome decreased in the follow-up. Triglycerides and HbA1c were the metabolic variables that improved significantly.

**Conclusions:**

The management of childhood obesity with an interdisciplinary intervention associated with continuous group educational support can significantly impact on clinical and metabolic changes. It is necessary to continue monitoring over time to prevent relapse.

La obesidad es un problema de salud pública que se ha catalogado como la epidemia del siglo XXI ^1^. Según el informe de la Organización Mundial de la Salud (OMS), en el 2016 se reportaron más de 340 millones de niños y adolescentes entre los 5 y los 19 años de edad con diagnóstico de sobrepeso u obesidad. La prevalencia mundial de obesidad infantil pasó del 1 % en 1975 al 6 % en niñas y el 8 % en niños en el 2016, lo cual corresponde a 124 millones de niños y adolescentes obesos en el mundo ^2^^,^^3^.

El perfil epidemiológico colombiano no es ajeno a la situación mundial de la obesidad infantil. Los resultados de la Encuesta Nacional de la Situación Nutricional en Colombia (ENSIN 2015) revelaron un aumento en el exceso de peso (que engloba sobrepeso y obesidad). En escolares de 5 a 12 años de edad, el exceso de peso pasó de 18,8 a 24,4 % según las estadísticas de 2010 y 2015, en tanto que, en adolescentes de 13 a 17 años, el exceso de peso aumentó de 15,5 a 17,9 %. Lo anterior significa que casi uno de cada cuatro niños y, aproximadamente, uno de cada cinco adolescentes en la población general colombiana tenía exceso de peso ^4^.

En Medellín, el estudio del Perfil de Seguridad Alimentaria y Nutricional, SAN 2015, evidenció que el 15,7 % de los niños y adolescentes de 5 a 17 años de edad presentaba sobrepeso y, el 5,6 %, obesidad, para un total de 21,3 % con exceso de peso ^5^.

La obesidad se asocia con un alto riesgo de diabetes mellitus, síndrome metabólico, dislipidemia, aterosclerosis, riesgo cardiovascular y muchas otras alteraciones endocrinas y metabólicas, así como en otros sistemas ^6^^-^^8^ que, si no se detectan a tiempo, pueden evolucionar de manera silente y progresiva hasta convertirse en complicaciones graves, deteriorar la calidad de vida y causar una gran mortalidad ^9^^,^^10^.

La obesidad en la infancia afecta no solamente el estado de salud, sino también la capacidad de integración social y aumenta la probabilidad de que los niños se conviertan en adultos obesos ^11^. Además, es un factor de riesgo cardiovascular independiente y aporta una gran carga a las diversas causas de mortalidad global. En este sentido, se ha demostrado que los adolescentes obesos presentan una tasa de mortalidad 30 % mayor en la edad adulta ^9^^,^^10^ y que más de dos terceras partes de los niños mayores de 10 años con obesidad seguirán siendo obesos en la adultez, con una disminución de la esperanza de vida de unos 5 a 10 años ^10^.

Dado que la obesidad es una condición grave que puede comenzar desde la infancia, los esfuerzos deben concentrarse en este grupo de edad, con el fin de prevenirla o, en su defecto, controlarla y evitar que progrese y se instaure con todas sus complicaciones y secuelas en la edad adulta ^12^^-^^15^.

En Colombia, se promulgó en el 2009 la Ley 1355, en la cual se definen la obesidad y las enfermedades crónicas no transmisibles asociadas con esta como una prioridad de salud pública, y se adoptan medidas para su control, atención y prevención. En el artículo 1 se establece

"[...] La obesidad como una enfermedad crónica de salud pública, la cual es causa directa de enfermedades cardiacas, circulatorias, colesterol alto, estrés, depresión, hipertensión, cáncer, diabetes, artritis, colon, entre otras, todos ellos aumentando considerablemente la tasa de mortalidad de los colombianos [...]" ^16^.

Para enfrentar con éxito la prevención de la obesidad infantil, es necesario contrarrestar el ambiente 'obesogénico' y adoptar hábitos saludables de estilo de vida mediante una acción coordinada y multisectorial.

Entre las recomendaciones de la OMS en el Informe de la Comisión del año 2017 para acabar con la obesidad infantil, se cuentan las siguientes ^17^.

- Emplear estrategias que promuevan la ingestión de alimentos sanos, reduzcan la de alimentos no saludables y bebidas azucaradas, e incentiven la actividad física durante los períodos cruciales de la vida: antes y durante la gestación, en la etapa de la lactancia, la infancia y la adolescencia.
- Ofrecer servicios de salud integrales para el control del peso corporal, que reúnan diversos componentes y se centren en la familia y en la modificación del estilo de vida. Se resalta la importancia de poner en marcha servicios adecuados para el control del peso

"[…] dirigidos a niños y adolescentes con sobrepeso u obesidad que reúnan diversos componentes (nutrición, actividad física y apoyo psicosocial), se centren en la familia y corran a cargo de equipos integrados por varios profesionales con formación y recursos adecuados, como parte de la cobertura sanitaria universal [...]". ^17^

En numerosos estudios se ha evaluado la efectividad de los programas multidisciplinarios con apoyo familiar para tratar el sobrepeso y la obesidad infantil ^11^^,^^15^^,^^18^^-^^20^. Sin embargo, la obesidad es una enfermedad crónica y mantener el índice de masa corporal (IMC) esperado para la edad después de una pérdida de peso inicial o de su estabilización representa el principal desafío ^19^^,^^21^.

En el Hospital Universitario de San Vicente Fundación, centro de referencia de pacientes de alta complejidad, atiende un número cada vez mayor de niños y adolescentes obesos que ingresan a través de diferentes servicios, como la consulta de endocrinología infantil, de nutrición, y de medicina física y rehabilitación. Muchos de ellos ya han recibido atención en otras instituciones sin obtener los resultados esperados, probablemente debido a la ausencia de un enfoque integral de esta enfermedad de origen multifactorial.

En nuestro medio, los programas de atención integral de la obesidad son insuficientes. En vista de la creciente prevalencia de esta condición, en el 2011, se creó el Programa de Obesidad Infantil del Hospital Universitario de San Vicente Fundación, el cual incorpora la intervención clínica multidisciplinaria a actividades educativas grupales dirigidas a los pacientes y sus familias, con el propósito de intervenir los factores condicionantes nutricionales y conductuales implicados en el desarrollo de la obesidad y de sus complicaciones.

Dado que en nuestro medio son pocos los estudios sobre el impacto de las intervenciones multidisciplinarias en la obesidad infantil, se adelantó un estudio para evaluar los cambios en los parámetros clínicos y metabólicos de una cohorte de pacientes después de su participación en el programa de atención integral en obesidad infantil del Hospital Universitario de San Vicente Fundación entre 2012 y 2015.

## Materiales y métodos

### Tipo de estudio y población

Se hizo un estudio retrospectivo, observacional, longitudinal y analítico, en una cohorte de pacientes atendidos en el Programa de Obesidad Infantil. El estudio se basó en la información registrada en las historias clínicas y en los formatos del programa.

La población de estudio estuvo conformada por pacientes atendidos en el Programa de Obesidad Infantil del Hospital Universitario de San Vicente Fundación entre el 2012 y el 2015, que atiende a niños y adolescentes con diagnóstico de obesidad exógena según los patrones de crecimiento de la OMS ^22^. Los pacientes eran remitidos al programa por profesionales de la salud de otras instituciones o de consulta particular, o ingresaban por solicitud de la familia o los cuidadores.

Los criterios de inclusión estipulaban la participación de pacientes entre los 6 y los 17 años, de ambos sexos y con diagnóstico de obesidad exógena, que tuvieran las siguientes condiciones:

- Puntaje Z del IMC entre +2 y +3 más, al menos, uno de las siguientes comorbilidades: síndrome metabólico [según los criterios del *National Cholesterol Education Program Adult Treatment Panel III* (NCEP-ATP III) modificados para niños y adolescentes] 23, dislipidemia [según el *NCEP Expert Panel on Cholesterol Levels in Children*], alteración del metabolismo de los carbohidratos [según la *American Diabetes Association* (ADA) ] 24 o hipertensión arterial [según el *National High Blood Pressure Education Program Working Group on High Blood Pressure in Children and Adolescents*] 25.
- Puntaje Z del IMC mayor o igual a +3 DE, con comorbilidades asociadas o sin ellas.

Se excluyeron del programa aquellos pacientes con alteraciones neurocognitivas, inasistencia a las actividades del programa por un período mayor de seis meses o farmacodependencia.

### Descripción de las actividades del programa

*Ingreso y atención clínica.* Los pacientes ingresaban al programa remitidos de las consultas de endocrinología pediátrica, de nutrición o de medicina física y rehabilitación, y luego eran remitidos a las demás especialidades (endocrinología pediátrica, nutrición clínica pediátrica, medicina física y rehabilitación, psicología, psiquiatría, trabajo social y terapia de familia).

Al ingreso, se solicitaban los siguientes exámenes de laboratorio: colesterol total, triglicéridos, cLDL, cHDL, glucemia en ayunas y transaminasas. Estas pruebas se repetían cada 3 a 6 meses, dependiendo del resultado. Además, se solicitaban exámenes complementarios según las comorbilidades de cada paciente, por ejemplo, hemoglobina A1c (HbA1c), 25-hidroxivitamina D, glucosa basal y dos horas poscarga, entre otros. Estos exámenes se ordenaban convencionalmente a lo largo del seguimiento en el programa y eran autorizados por las aseguradoras, las cuales determinaban en qué laboratorios se harían según su red de atención.

Durante el seguimiento, los pacientes recibían atención por las especialidades tratantes cada tres a seis meses o más frecuentemente según su condición clínica. La duración del programa era mínimo de un año, con posibilidad de extenderse por más tiempo si las condiciones del paciente lo requerían.

En cada una de las consultas, se brindaba educación individualizada al paciente y a su familia sobre estilos de vida saludable y riesgo de comorbilidades. 

*Junta médica.* Los pacientes con evolución tórpida eran analizados en una junta médica que incluía la participación de especialistas en psiquiatría infantil, terapia de familia, medicina física y rehabilitación, nutrición clínica pediátrica y endocrinología. Estas juntas se realizaban cada mes, aproximadamente.

*Componente educativo del programa.* Se desarrolló una intervención educativa dirigida a los pacientes y a sus padres o cuidadores, con base en la metodología cognitivo-conductual y según los lineamientos del programa "Niños en movimiento" (Universidad Autónoma de Barcelona) ^26^ para niños de 6 a 12 años de edad, previa autorización y entrenamiento por parte de los autores de dicho programa. La intervención se adoptó con modificaciones según las condiciones socioculturales propias de la población intervenida. Se realizaron ocho sesiones quincenales de 90 minutos de duración, en paralelo para padres y niños (máximo diez pacientes por sesión), a cargo de nutricionistas, psicólogas y educadores físicos.

Cada sesión incluía secciones teóricas y prácticas, con ejercicios didácticos y experienciales para el aprendizaje de contenidos que incluían temas como la alimentación saludable (importancia del desayuno, clasificación de los alimentos, diversificación de la alimentación), manejo de la alimentación a deshoras, actividad física (actividad física en la vida cotidiana, ejercicio programado), horas frente a las pantallas, publicidad engañosa, autocontrol, imagen corporal, comunicación, asertividad y autoestima. Además, se hicieron talleres prácticos interactivos de cocina saludable para padres y niños, de una hora de duración, orientados por nutricionistas y un chef.

*Actividad física.* A todos los pacientes se les recomendó el ejercicio y se les ordenaron doce sesiones de rehabilitación cardiaca, según su tolerancia a la actividad física y su evolución a lo largo de las sesiones, y también, se les dieron indicaciones específicas para optimizar el ejercicio en casa. La rehabilitación cardíaca se realizaba dos veces a la semana en sesiones de una hora de duración, en las cuales se monitorizaban los signos vitales y se tomaban las medidas antropométricas; se hacían 5 minutos de calentamiento y, luego, 30 a 45 minutos de ejercicio aeróbico (en banda sin fin, bicicleta estacionaria, actividades deportivas o baile usando la consola Wii), que aumentaba progresivamente hasta lograr una intensidad moderada a fuerte según la escala de evaluación subjetiva de cansancio (escala de Borg) ^27^ y la respuesta hemodinámica durante el ejercicio. Hubo monitorización continua con telemetría del trazado electrocardiográfico y oximetría de pulso durante todo el ejercicio y la sesión finalizaba con cinco minutos de ejercicios de relajación.

### Recolección de la información

La fuente de información para el estudio provino de los formatos de registro del programa de obesidad (previamente diligenciados) y de la historia clínica electrónica de cada paciente. No se calculó el tamaño de la muestra, ya que se incluyeron todos los pacientes atendidos en el Programa de Obesidad Infantil entre enero de 2012 y diciembre de 2015 que hubieran tenido un seguimiento, por lo menos, de seis meses.

En cada una de las consultas con los especialistas, se registraba la información en un formato diseñado con este fin que se diligenciaba convencionalmente en el programa de obesidad, e incluía las variables de edad, sexo, datos antropométricos (peso, talla, puntaje Z del IMC, perímetro abdominal), los criterios de síndrome metabólico según el NCEP-ATP III modificados para niños y adolescentes ^23^, la clasificación del perímetro abdominal (según percentil) ^28^, los resultados de laboratorio (colesterol total, triglicéridos, cLDL, cHDL, glucosa en ayunas), y la presión arterial sistólica y diastólica.

Se evaluaron, también, variables como la actividad física semanal y el tiempo frente a las pantallas determinado a partir de las preguntas al paciente y su familia, la asistencia a la rehabilitación cardiaca y variables sociales (tipología familiar, eventos vitales y escolaridad) evaluados por el terapeuta de familia.

La definición de síndrome metabólico se ajustó a los criterios del NCEP-ATP III modificados para niños y adolescentes ^23^, es decir, se diagnosticaba si se cumplían tres o más criterios positivos de los siguientes:

1. perímetro abdominal mayor o igual al percentil 90 para la edad y el sexo, según los valores de referencia de la *Third National Health and Nutrition Examination Survey* (NHANES III) ^28^;
2. presión arterial sistólica o diastólica mayor o igual al percentil 90 para la edad y el sexo, y el percentil de talla según las tablas de presión arterial del *Fourth National High Blood Pressure Education Program Working Group on High Blood Pressure in Children and Adolescents*^25^;
3. concentración plasmática de triglicéridos mayor o igual a 110 mg/dl;
4. concentración plasmática de colesterol HDL menor o igual a 40 mg/dl, y
5. glucemia en ayunas mayor o igual a 100 mg/dl (recomendaciones de la *American Diabetes Association,* ADA) ^24^.

Se decidió utilizar los criterios del NCEP-ATP III modificados para niños y adolescentes ^23^ porque los de la *International Diabetes Federation* (IDF) ^29^ tienen límites más altos comparados con los establecidos en el NCEP-ATPIII, modificados ^23^, por lo que con ellos se detectan menos casos ^30^^,^^31^. Además, los puntos de corte para los triglicéridos y la presión arterial distan significativamente de los límites superiores postulados por las guías de riesgo cardiovascular en niños y adolescentes ^32^.

Estos criterios permiten analizar los valores de presión arterial por percentil según edad, sexo y talla, lo que es más adecuado en el enfoque pediátrico ^23^, en tanto que los criterios de la *International Diabetes Federation* (IDF) incluyen los valores absolutos de presión arterial (≥130/85 mm Hg) y de triglicéridos (≥150 mg/dl) tomados de las definiciones de síndrome metabólico en adultos ^29^.

Además, en el estudio de Agudelo, *et al.*^33^, en adolescentes de Medellín, se encontró que, al comparar distintos tipos de criterios de síndrome metabólico, los de Ford ^23^ fueron los más apropiados para evaluar el riesgo cardiovascular en las condiciones específicas de nuestra población. Con estos criterios se logra una detección más temprana de pacientes con riesgo de diabetes y enfermedad cardiovascular ^33^.

En cada evaluación de seguimiento se registraban los resultados de laboratorio en el formato de recolección de datos. Estos exámenes se hacían en los laboratorios de la red de atención de cada aseguradora. La información que no estaba disponible en los formatos de registro del programa se extrajo de la historia clínica.

El peso se tomó utilizando una báscula Weigh-Tronix™ (sensibilidad de 0,01 kg), la talla con un estadiómetro JANDAC™ (sensibilidad de 1 mm) y el perímetro abdominal con una cinta métrica antropométrica SECA™ (sensibilidad de 1 mm). Dichos instrumentos fueron calibrados por el Servicio de Bioingeniería del hospital.

Las mediciones de la talla estuvieron a cargo de las endocrinólogas pediátricas del hospital. En la consulta se hacían dos mediciones de talla y, si había discrepancias, se tomaba una tercera medición para registrar el promedio. El peso se tomaba sin ropa y era registrado por enfermeras previamente entrenadas. La medición de la circunferencia de cintura estuvo a cargo de las endocrinólogas según la técnica del manual de procedimientos antropométricos de la encuesta NHANES ^34^ y se interpretó según los percentiles presentados por Cook, *et al.*^28^.

El cálculo del puntaje Z del IMC se hizo con el programa de la OMS Anthro Plus de distribución gratuita ^35^. Las técnicas de registro de la información utilizadas por los profesionales en el programa, estaban estandarizadas.

### Análisis estadístico

Se determinó si las variables cuantitativas provenían de una población con distribución normal (prueba de Kolmogorov-Smirnov: p>0,05). Las variables numéricas con distribución normal se expresaron en medias y desviación estándar, y aquellas con distribución diferente a la normal, en medianas y rangos intercuartílicos. Las variables categóricas se presentan en números absolutos y relativos.

 Con el fin de controlar el sesgo generado por las diferencias en la duración del seguimiento durante el programa, se hizo un análisis estratificado según los meses de seguimiento de la variable IMC (puntaje Z) y de las variables metabólicas (glucemia, hemoglobina A1c, triglicéridos, c-HDL, colesterol total, c-LDL y los criterios agrupados de síndrome metabólico). 

Para determinar las diferencias entre los valores de las variables al ingreso y al final de la intervención, se utilizaron la prueba t de Student en el caso de la distribución normal y la prueba de Wilcoxon para las de distribución no normal. La prueba de x^2^ se empleó para determinar la diferencia en la proporción de las variables cualitativas, con un valor de p<0,05 como significativo. La información se almacenó y procesó en el programa estadístico PASW Statistics 18 (SPSS 18™).

### Consideraciones éticas

Según el Ministerio de Salud de Colombia, Resolución 008430 de octubre de 1993, artículo 11, esta investigación se consideró sin riesgo por ser de tipo observacional retrospectivo y porque los datos se extrajeron de las historias clínicas y de los registros del programa de obesidad. En todos los casos se protegió la identidad de los individuos incluidos en el estudio, pues la base de datos se depuró antes de acceder a ella para excluir la información personal (nombres y documentos de identidad).

El estudio fue aprobado por el Comité de Ética de la Investigación del Hospital Universitario de San Vicente Fundación.

## Resultados

Se incluyeron 53 niños y adolescentes que ingresaron al programa de obesidad del Hospital Universitario de San Vicente Fundación, entre enero de 2012 y abril de 2015. El tiempo promedio de seguimiento fue de 18 ± 6 meses. El 15 % de los niños tuvo un seguimiento de 12 meses o menos, y el 30 %, de 31 a 36 meses, en tanto que el 52,8 % tuvo un seguimiento, por lo menos, de dos años (cuadro 1).

**Cuadro 1:** Características sociodemográficas de la población de estudio (n=53)

Edad (años), (X ± DE)	11 ± 2
Sexo	n (%)
Masculino	28 (52,8)
Tiempo seguimiento (meses), (X ± DE)	18 ± 6
Seguimiento (meses)	n (%)
≤12	8 (15,1)
13 a 18	8 (15,1)
19 a 24	9 (17)
25 a 30	9 (17)
31 a 36	19 (35,8)
Tipología familiar	n (%)
Nuclear	23 (44)
Extensa	18 (34)
Monoparental	6 (12)
Reconstituida	5 (10)

(X ± DE): promedio ± desviación estándar

La edad promedio fue de 11 ± 2 años, siendo la edad mínima de 6 y la máxima de 16 años. El 39,6 % era adolescente y, el 52,8 %, de sexo masculino.

Con respecto a la tipología familiar, se encontró que solo el 44 % de los pacientes residía con ambos padres y, en el 56 % de los casos restantes, la composición familiar respondía a otras modalidades (12 %, monoparental; 34 %, familia extensa; 10 %, reconstituida).

El 34 % de los pacientes reportó un familiar con obesidad o sobrepeso.

### Variables antropométricas

Considerando que el principal indicador antropométrico de obesidad es el IMC, se comparó la media del puntaje Z al ingreso (2,75 ± 0,58) con el de la última evaluación (2,32 ± 0,63) y se encontró una disminución significativa en todos los pacientes, independientemente del tiempo en el programa (prueba t de Student para muestras relacionadas: p=0,000; IC_95_% 0,27-0,57) (cuadro 2).

**Cuadro 2:** Medidas de tendencia central del IMC (puntaje Z) al ingreso *Vs.* última evaluación según grupos de edad y tiempo de seguimiento

Rangos de edad (años)	Ingreso	Última medición			
	IMC (puntaje Z)*	IMC (puntaje Z)*	Diferencia de medias	IC_95%_	p**
6 a 9 (n=16)	2,88 ± 0,71	2,42 ± 0,73	0,46	0,19 a 0,72	0,002
10 a 13 (n=20)	2,72 ± 0,63	2,26 ± 0,54	0,46	0,19 a 0,72	0,002
14 a 16 (n=17)	2,67 ± 0,38	2,31 ± 0,67	0,36	0,05 a 0,66	0,024
**Seguimiento (meses)**	IMC (puntaje Z)*	IMC (puntaje Z)*	**Diferencia de medias**	**IC_95%_**	p**
≤12	2,61 ± 0,65	2,38 ± 0,81	0,23	-0,03 a 0,49	0,077
13 a 18	2,94 ± 0,28	2,40 ± 0,41	0,54	0,19 a 0,87	0,008
19 a 24	3,00 ± 0,54	2,61 ± 0,67	0,39	-0,07 a 0,84	0,087
25 a 30	2,65 ± 0,49	2,32 ± 0,63	0,33	0,00 a 0,64	0,046
31 a 36	2,65 ± 0,69	2,12 ± 0,60	0,53	0,19 a 0,86	0,004
Total (n=53)	2,75 ± 0,58	2,32 ± 0,63	0,43	0,27 a 0,57	0,000

IMC: índice de masa corporal (puntaje Z)

* (X ± DE)

** t de Student de muestras relacionadas

Significación estadística: p<0,05

Al evaluar el cambio en el IMC discriminando por subgrupos de edad, se encontró que todos registraron una reducción significativa del puntaje Z, siendo similar el cambio en niños de 6 a 9 años y de 10 a 13 años. En los adolescentes entre los 14 y los 16 años, hubo menos disminución, pero aun así fue estadísticamente significativa (cuadro 2).

Al discriminar por subgrupos según la duración del seguimiento, se observó que hubo disminución del puntaje Z del IMC en todos los grupos, con una reducción estadísticamente significativa en los grupos con seguimiento de 13 a 18 meses y con los de más 24 meses (cuadro 2).

El 44 % de los pacientes bajó el puntaje Z del IMC en menos de 0,5, el 46 % en 0,5 a 1,0 y el 9,3 % lo redujo en más de 1,0. La media de reducción del puntaje Z del IMC para toda la población atendida en este estudio fue de 0,43.

Al comparar la variación del puntaje Z (IMC) entre el ingreso y la última evaluación, la proporción de pacientes en quienes disminuyó el IMC fue de 79,2 % y la de aquellos en quienes aumentó fue de 20,8 %.

**Figura 1:**
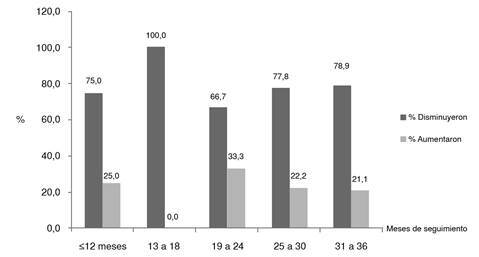
Proporción de pacientes que disminuyeron su IMC *Vs.* los que lo aumentaron entre el ingreso y la última evaluación (enero de 2012 a abril de 2015)

En todos los pacientes que permanecieron entre 13 y 18 meses en el programa, el IMC disminuyó. Los pacientes con un seguimiento de más de 18 meses registraron una proporción de aumento del IMC en el tiempo, aunque el número de quienes presentaron este aumento disminuyó progresivamente a lo largo del seguimiento, con tendencia a estabilizarse (figura 1).

**Figura 2:**
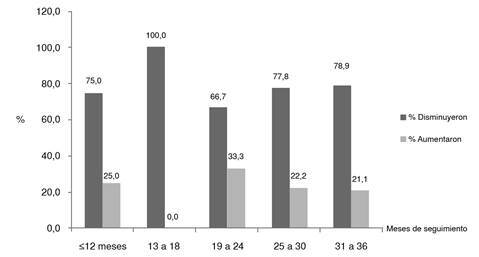
Proporción de niños según la clasificación del IMC (puntaje Z) en el ingreso *Vs.* la última evaluación (enero de 2012 a abril de 2015)

Al comparar la clasificación del IMC (puntaje Z) en el ingreso con la de la última evaluación en todos los participantes en el programa, se observó que la proporción de niños obesos con un IMC (puntaje Z) de más de 3 descendió de forma significativa del 33,4 al 14,6 % (x^2^, p=0,000), y se logró que el 25 % de la población descendiera al rango de sobrepeso en la última evaluación (figura 2).

### Cambios en los componentes del síndrome metabólico y la HbA1c

Al ingreso, el 37 % de los pacientes cumplían los criterios diagnósticos del síndrome metabólico (tres o más criterios positivos), cifra cercana a la encontrada en el último control (32 %). Sin embargo, la proporción de pacientes que tenía cuatro o cinco criterios positivos, bajó del 15 al 2 % y, específicamente en aquellos que tuvieron más de 18 meses de seguimiento, esta proporción pasó de 18,9 a 0 %.

El 85 % de los pacientes tuvo control de glucemia en ayunas en la última evaluación clínica, y en ninguno hubo diferencias estadísticas en el tiempo. Sin embargo, en los que tuvieron un seguimiento menor de 12 meses, sí se observó una disminución significativa del valor absoluto de la glucemia.

De los 53 pacientes evaluados, tres (5,6 %) tenían un valor de glucemia en ayunas en el rango de prediabetes al ingresar. En la última evaluación, en dos de esos tres pacientes se normalizó el valor de la glucemia en ayunas, por lo que ya no tenían diagnóstico de prediabetes.

En el 75 % de todos los pacientes se registró la glucosa dos horas poscarga y se encontró que su valor fue superior a 140 mg/dl (prediabetes) en dos de ellos, pero no se observó progresión a diabetes de tipo 2; lo que sí se encontró es que mejoraron su HbA1c de 5,7 a 5,1 % y de 6,4 % a 5,4 %.

En 42 (79 %) de los 53 de los pacientes se midió la HbA1c al ingreso y en la última evaluación. La mediana del inicio pasó de 5,5 % (rango intercuartílico, RIC: 5,2 - 5,9 %) a 5,3 % en el último control (RIC: 5,1-5,4 %) (prueba de Wilcoxon: p=0,05). El rango de distribución de la HbA1c fue más estrecho al final y no pasó del límite de prediabetes. Al evaluar el cambio en la HbA1c en el subgrupo con seguimiento de más de 31 meses, esta pasó de 5,6 a 5,2 % (prueba Wilcoxon, p=0,016).

En el ingreso, el 64 % de los pacientes tenía valores normales (<5,6 %) de HbA1c ^24^; en el último control, este porcentaje mejoró a 92 %.

En cuanto al perfil lipídico, se observó disminución del colesterol total y de las LDL, con mejoría de las HDL; sin embargo, estos cambios no fueron significativos (cuadro 3).

**Cuadro 3:** Perfil lipídico y HbAIc en el ingreso al programa y última medición según tiempo en el programa

Laboratorio	Seguimiento (meses)	Ingreso Me (RIC)	Última medición Me (RIC)	p**
Glucemia (mg/dl)	≤12	88,5 (83-92)	80 (72-90)	0,046
	13 a 18	88,0 (82-94)	86,5 (82-92)	0,893
	19 a 24	82,5 (79-97)	83 (77-86)	0,674
	25 a 30	90 (83-91)	87 (85-89)	0,612
	31 a 36	86 (81-88)	86 (79-91)	0,679
	Total	86 (82-90)	86 (79-89)	0,199
HbA1c (%)	≤12	N	N	N
	13 a 18	5,6 (5,2 - 6,1)	5,4 (5,2-5,)	0,223
	19 a 24	5,5 (5,1-5,8)	5,1 (5,1-5,6)	0,273
	25 a 30	5,3 (5,9-5,6)	5,5 (5,1-5,5)	0,686
	31 a 36	5,6 (5,4-5,9)	5,2 (4,9-5,5)	0,016
	Total	5,5 ( 5,2-5,9)	5,3 (5,1-5,4)	0,05
Colesterol total (mg/dl)	≤12	187 (149-210)	169 (156-200)	0,866
	13 a 18	172 (147-189)	149 (138-198)	0,183
	19 a 24	176 (161-196)	171 (153-200)	0,956
	25 a 30	166 (130-181)	144 (123-193)	0,813
	31 a 36	157 (141-170)	154 (135-167)	0,157
	Total	164 (144,5-187)	158 (141,5-181)	0,13
Triglicéridos (mg/dl)	≤12	123 (85-163)	129 (99-136)	0,499
	13 a 18	105 (69-182)	68 (65-96)	0,208
	19 a 24	111 (86-190)	90 (83-176)	0,343
	25 a 30	113 (80-141)	74 (64-92)	0,066
	31 a 36	131 (89-183)	99 (77-149)	0,033
	Total	114 (85,5-175,5)	96 (72-138)	0,033
Colesterol LDL (mg/dl)	≤12	107 (84-150)	100 (92-131)	0,866
	13 a 18	99 (83-119)	99 (78-134)	0,674
	19 a 24	108 (85-136)	106 (92-137)	0,956
	25 a 30	79 (66-118)	84 (59-123)	0,44
	31 a 36	94 (81-100)	92 (78-110)	0,528
	Total	97 (80,2-119)	94 (81,2-122)	0,75
Colesterol HDL (mg/dl)	≤12	42 (36-54)	47 (41-52)	0,599
	13 a 18	46 (36-52)	42 (30-52)	0,31
	19 a 24	44 (35-48)	43 (38-48)	0,734
	25 a 30	44 (40-50)	44 (43-59)	0,121
	31 a 36	40 (30-44)	43 (32-54)	0,07
	Total	42 (35,5-47)	44 (40-50,7)	0,144

** Wilcoxon, significación: p<0,05

Me (RIC): mediana (rango intercuartílico)

Significación: p<0,05

Con respecto a los valores de los triglicéridos, se observó que en todos los participantes hubo una disminución entre el ingreso y el último control, pues la mediana de triglicéridos en el ingreso fue de 114 mg/dl (RIC: 85,5-175,5), en tanto que en el último control, esta fue de 96 mg/dl (RIC: 72-138), con una diferencia estadísticamente significativa (prueba de Wilcoxon: p=0,03). Al discriminar por duración del seguimiento, se encontró una reducción de los triglicéridos en casi todos los subgrupos, la cual fue estadísticamente significativa en el grupo con más de 31 meses de seguimiento.

El perímetro abdominal fue mayor del percentil 90 al ingreso en el 90,6 % de los pacientes y en el 84,9 % en el último control (prueba de x^2^, p=0,02).

## Discusión

La incidencia de la obesidad en niños y adolescentes sigue incrementándose de manera alarmante, por lo que su prevención e intervención son una prioridad en salud pública ^4^, que ha llevado a plantear varios tipos de aproximación, incluidas tanto las intervenciones individuales como las grupales mediante programas multidisciplinarios. En diversos estudios se han evaluado múltiples intervenciones ^36^ que van desde las basadas exclusivamente en un aspecto como la alimentación saludable ^37^ o el ejercicio ^38^^,^^39^ hasta aquellas en que se combinan varios elementos: educación y comportamiento ^40^, alimentación y actividad física ^41^^,^^42^, alimentación y educación ^37^, y, de forma más completa, manejo nutricional, conductual y actividad física ^43^^-^^48^.

En el presente estudio se analizó un programa con enfoque integral, consistente en la evaluación multidisciplinaria de diversas especialidades clínicas, complementada con educación a los pacientes y a los cuidadores en torno a cambios en el estilo de vida, la alimentación saludable, la actividad física y el manejo emocional.

Gracias a esta intervención integral, se logró una disminución significativa del promedio del IMC (puntaje Z) al comparar el ingreso con el último control. Además, la proporción de pacientes con un puntaje Z del IMC mayor de 3 pasó del 33,4 al 14,6 %, en tanto que, en el 25 %, el diagnóstico de obesidad cambió a sobrepeso.

Dado que no fue posible atender todos los pacientes durante el mismo número de meses, se tuvieron que estratificar los cambios en el IMC y en las variables metabólicas según el tiempo de seguimiento. A pesar de esta variabilidad en el tiempo de seguimiento, la mitad de ellos lograron contar con un acompañamiento por más de dos años.

La reducción obtenida del IMC en el 79,25 % de los pacientes es similar a lo reportado por Reinehr, *et al.*^48^, en un estudio multicéntrico nacional con programas de tratamiento de obesidad infantil en Alemania (42 centros ambulatorios con 1.041 pacientes), en el cual se reportó una reducción del IMC en 64 % de los pacientes a lo largo del seguimiento. Ambos estudios demuestran el impacto positivo de los programas de intervención en este grupo de población.

Además, en el estudio de Reinehr, *et al.*^48^, el 11 % de los pacientes redujo su IMC en 0,5 DE, el 23 % entre 0,2 y 0,5 DE, el 30 % menos de 0,2 DE y el 21 % lo aumentó. Según los resultados del presente estudio, el 44 % bajó 0,5 DE del IMC, el 46 % 0,5-1,0 DE y el 9,3 % bajó más de 1 DE. Las diferencias entre los resultados de uno y otro estudio se podrían explicar por el hecho de que, en el presente, el modelo de intervención fue más intensivo y el grupo de pacientes, al ser más pequeño, fue menos heterogéneo. En el estudio multicéntrico en Alemania, 114 de 1.041 pacientes redujeron más de 0,5 DE su IMC, mientras que, en el presente, 24 de 53 pacientes obtuvieron esa misma reducción.

El puntaje Z del IMC promedio de toda la población atendida en nuestro programa logró reducirse en 0,43, cambio similar al encontrado en otro estudio de Reinehr, *et al.*^46^, en el que se redujo 0,40 al año y 0,41 a los dos años, entre el inicio y el final de la intervención.

Nuestros resultados fueron similares a los de Hampl, *et al.*^49^, quienes encontraron que cambio en la media del puntaje Z del IMC fue de -0,11 a los 24 meses de seguimiento, y en el de Reinehr, *et al.,* en el cual dicho cambio fue de -0,20 durante el mismo tiempo de seguimiento ^48^, lo que corrobora las bondades de estos programas de intervención en la obesidad infantil.

La recomendación de la *International Obesity Task Force* (IOTF) de 2016 es una disminución de 0,2 en el puntaje Z del IMC como una medida estandarizada de efectividad del tratamiento de la obesidad 50. Según este reporte, la reducción de 0,15 a 0,25 en dicho puntaje se asocia con una mejoría en los resultados cardiometabólicos, siendo una reducción de 0,2 equivalente a una disminución de 5 % en el peso corporal ^50^.

En el presente estudio, se logró reducir este puntaje en 0,43, resultado que superó el límite internacional estandarizado de efectividad propuesto por la IOTF. Ello se relacionaría con el enfoque multidisciplinario que se mantuvo a lo largo del programa de obesidad, en el cual se incluyeron, no solo las áreas clínicas (endocrinología, nutrición infantil, fisiatría, psiquiatría infantil), sino también las psicosociales (psicología, trabajo social y terapia de familia), para mejorar el cumplimiento e intervenir otras enfermedades asociadas. Además, el trabajo educativo grupal incluyó el componente nutricional, el de actividad física y el emocional. La educación y el enfoque multidisciplinario pueden contribuir a mejorar los resultados con base en las recomendaciones de diferentes guías y revisiones en torno al tratamiento de la obesidad ^51^^-^^54^, lo cual justifica continuar con la implementación de este tipo de programas.

En cuanto al efecto en los cambios metabólicos, se encontró una disminución en la proporción de pacientes que cumplía con los criterios del diagnóstico de síndrome metabólico. Si bien el porcentaje de diagnóstico de este síndrome no cambió mucho, sí disminuyó el número de criterios positivos y el riesgo metabólico acumulado. La proporción de pacientes que tenía cuatro o más criterios positivos (con mayor acumulación de riesgo metabólico) bajó del 15 al 2 %, lo que sugiere que se presentó una redistribución de la proporción que presentaba un gran número de criterios, es decir, los que antes cumplían con cuatro o cinco criterios positivos pasaron a tener tres o cuatro, lo que demuestra una reducción del riesgo metabólico.

En publicaciones recientes, se ha planteado la necesidad de enfocar el síndrome metabólico, no a partir de la dicotomía de cumplir con los criterios del diagnóstico o no, sino a partir de la evaluación del riesgo metabólico como un *continuum* según el número de criterios acumulados ^55^^-^^59^.

Algunos autores han propuesto puntajes continuos para el síndrome metabólico *(continuous Metabolic Syndrome scores,* cMetS), obtenidos de la suma de los puntajes Z de las variables individuales del síndrome metabólico, con el fin de mejorar el diagnóstico en niños y adolescentes. Un puntaje mayor del síndrome metabólico indicaría un perfil metabólico menos favorable ^55^^-^^59^.

En el presente estudio, independientemente del porcentaje de pacientes con diagnóstico de síndrome metabólico (tres criterios positivos), se encontró que la proporción con un mayor compromiso metabólico por cumplir con cuatro o más criterios positivos acumulados, disminuyó. Probablemente, si se hubiera aplicado el método de los cMets a estos pacientes, se hubiera encontrado también una reducción cuantitativa del riesgo; sin embargo, este análisis no estaba contemplado en la metodología del estudio, ya que no se contaba con las tablas de referencias por desviaciones estándar para las variables metabólicas propias de nuestra población.

En cuanto a la evaluación individualizada de los criterios de impacto metabólico y cardiovascular, Reinehr, *et al.,* encontraron una mejoría significativa de la presión arterial, la glucemia en ayunas y el perfil lipídico ^48^, similar a lo encontrado en el presente estudio, en el cual se evidenció una disminución significativa de los triglicéridos y de la HbA1c.

Al analizar específicamente la HbA1c de los pacientes del programa, se evidenció que la proporción de niños con valores anormales disminuyó entre el ingreso y el último control ^60^. En el 79 % de los pacientes del programa, la mediana de la medición de la HbA1c en el ingreso fue de 5,5 % (RIC: 5,2-5,9 %) y en la última evaluación estos valores bajaron a 5,3 % (RIC: 5,15,4 %). Según estos resultados, se observa inicialmente un rango de HbA1c correspondiente a una prediabetes (ADA: 5,7-6,4 %) ^60^. Al estratificar por tiempo de seguimiento, se encontraron diferencias estadísticas en dicha disminución solo en los pacientes con un seguimiento de más de 31 meses, lo que refleja indirectamente la mejoría del perfil del metabolismo de la glucosa en los sujetos con más tiempo de seguimiento en el programa. Los demás subgrupos en esta categoría no evidenciaron cambios significativos, pero no se descarta que haya sido por efecto del tamaño de la muestra.

Otro de los cambios metabólicos que mejoró significativamente fue la mediana de los triglicéridos, la cual disminuyó entre el inicio del programa y la última evaluación en todos los pacientes. Esto refleja cambios dinámicos, no solo en el aspecto metabólico de la obesidad, sino también en la calidad de la alimentación, como se ha reportado en la literatura especializada ^20^^,^^61^. Cabe anotar que la orientación clínica y la educación nutricional se ajustaron a las guías de riesgo cardiovascular en niños y adolescentes, las cuales establecen que en pacientes con niveles de triglicéridos elevados, la reducción de la ingestión de carbohidratos simples y la pérdida de peso están asociadas con disminución de los niveles de triglicéridos (evidencia de grado B) ^32^.

En cuanto al tiempo de seguimiento, el promedio de atención fue de 18 ± 6 meses, pero hasta el 30 % de los pacientes logró permanecer entre 31 y 36 meses. En estudios previos se ha hecho seguimiento de 24 meses ^47^ y 13 meses ^46^, y en otros, el seguimiento ha sido inferior, entre 6 y 12 meses ^36^. Con respecto a la permanencia en el programa, si se toma el límite de 24 meses para comparar con otros estudios, se evidencia que el 47,2 % no logró tener seguimiento de más de 24 meses. Aun así, dicho porcentaje es menor al reportado por Serra, *et al.*^62^, que informaron que el 78,7 % no completó el seguimiento de los 24 meses. La falta de cumplimiento del programa y, en mayor medida, el hecho de que las aseguradoras no enviaran a sus afiliados al programa debido a los cambios de contratación, fueron las principales causas de que el 47 % de los pacientes no continuara en el programa por más de dos años.

Una mayor duración de la intervención multidisciplinaria podría ayudar a atenuar el riesgo de recaída, lo que en parte explicaría el que algunos de los resultados del programa hayan sido más favorables que los obtenidos con un tiempo de seguimiento menor ^36^.

Aun así, en los estudios con periodos muy cortos de seguimiento, se han logrado resultados favorables al inicio, pero no podría asegurarse si en el largo plazo existiría un riesgo de recaída. De hecho, se ha visto que algunos programas de seguimiento a corto plazo podrían tener un efecto de rebote, por lo que se recomiendan seguimientos mayores de un año ^63^. Este riesgo de recaída es el mayor reto en el manejo de la obesidad ^53^^,^^54^.

En el presente estudio, los niños con disminución del IMC fueron más que los que registraron un aumento, independientemente del tiempo de seguimiento. Después de los 18 meses, la proporción que se acercó al IMC esperado para la edad aumentó progresivamente, con tendencia a estabilizarse luego de los 25 a 31 meses, lo que sugiere la importancia del seguimiento. Sin embargo, se requieren estudios adicionales para conocer la evolución de los pacientes con un seguimiento a más largo plazo. En el programa evaluado, no se pudo hacer un acompañamiento estructurado a todos los pacientes por más de 36 meses, ya que de ahí en adelante no consultaron o no pudieron hacerlo por falta de convenio con la aseguradora.

En estudios recientes, se destaca la importancia de un mayor seguimiento en los programas de obesidad infantil, tal como lo evidencia el metaanálisis publicado por van Der Heijden, *et al.*^21^, en el que se plantea que el tratamiento continuado tiene un efecto estabilizador en el puntaje Z del IMC. Considerando la magnitud del problema de la obesidad infantil, este hallazgo resalta la necesidad de implementar estrategias de mantenimiento de la mejoría del IMC ^21^. Además de los cambios antropométricos, se debería tener en cuenta la composición corporal para conocer con precisión las variaciones en el porcentaje de masa grasa y masa magra ^64^. Una limitación del presente estudio fue no incluir la evaluación de la composición corporal, pues su diseño era retrospectivo y no se contaba con dicha información. Por lo tanto, no se pudo establecer con exactitud la magnitud del cambio en la composición corporal en cuanto a tejido graso y magro.

Por último, es importante analizar que hubo un porcentaje de 20,2 % de pacientes que no mejoraron su IMC. Entre los factores que influyen en que no se logre la mejoría esperada, resalta el cumplimiento del programa. Se observó que parte de estos pacientes tenían como factores de riesgo los trastornos psiquiátricos (trastorno de ansiedad, trastorno por atracones, depresión), mientras que otros tenían poco apoyo familiar, inclusive, limitaciones económicas o logísticas para asistir a todas las actividades del programa.

Asimismo, además de los problemas de cumplimiento, y a pesar de que está demostrada la eficiencia de las intervenciones basadas en la alimentación, el ejercicio y la terapia conductual, especialmente cuando los padres están involucrados, el impacto en la pérdida de peso de estos programas es moderado; es más efectivo en los niños más pequeños y con menor grado de obesidad ^53^^,^^54^. Reinehr, *et al.,* resaltan que las fallas en la reducción de peso no deben atribuirse solo a falta de motivación y voluntad de cambio, sino, también, al sustrato genético que predispone a la obesidad; además, influyen los cambios hormonales adaptativos que implican reducción de la tasa metabólica basal y aumento del apetito durante el seguimiento. Por ello, Reinehr, *et al.,* plantean que la única explicación no puede ser la falta de cumplimiento del paciente y su familia, y que se requieren más estudios multicéntricos de calidad y con seguimiento a largo plazo para mejorar las estrategias de los programas contra la obesidad ^53^^,^^54^.

Otra limitación del programa evaluado fue la imposibilidad de hacer el seguimiento de todos los pacientes por el mismo número de meses, por lo cual se debieron estratificar los cambios en el IMC y en los resultados metabólicos según el tiempo de seguimiento. Una limitación adicional fue la restricción del número de pacientes analizados por las dificultades en el seguimiento completo, debidas a la intermitencia en la contratación de las aseguradoras, así como en el cumplimiento del programa por parte de los participantes.

Debe mencionarse, igualmente, que no fue posible hacer los estudios metabólicos con el mismo método y en el mismo laboratorio, dado que estos exámenes se hacían en los laboratorios de la red de atención de la aseguradora de cada paciente, lo cual puede generar diferencias en los resultados según los métodos utilizados.

Por todo ello, se recomienda hacer estudios posteriores de tipo analítico y prospectivo con un mayor número de pacientes, para comparar diferentes tipos de intervenciones y hacer un seguimiento estandarizado de los parámetros metabólicos y de su evolución en el tiempo.

En conclusión, el manejo de la obesidad infantil requiere de intervenciones multidisciplinarias con varias dimensiones acompañadas de actividades educativas grupales continuadas y con apoyo de la familia, que incorporen el componente nutricional, el emocional y el de actividad física. Estas intervenciones integrales pueden mejorar la evolución del IMC y los componentes de riesgo metabólico si se aplican de manera estructurada durante el tiempo apropiado para disminuir o eliminar el riesgo de recaídas.
